# A Ketoconazole Susceptibility Test for *Malassezia pachydermatis* Using Modified Leeming–Notman Agar

**DOI:** 10.3390/jof4040126

**Published:** 2018-11-16

**Authors:** Bo-Young Hsieh, Wei-Hsun Chao, Yi-Jing Xue, Jyh-Mirn Lai

**Affiliations:** 1 Lugu Township Administration, Nanto County 55844, Taiwan; Qwerkboy@gmail.com; 2 College of Veterinary Medicine, National Chiayi University, No. 580, XinMin Rd., Chiayi City 60054, Taiwan; asdzxc0523@gmail.com; 3 Department of Hospitality Management, WuFung University, Chiayi City 60054, Taiwan; whchao@wfu.edu.tw

**Keywords:** disk diffusion, minimal inhibitory concentration, *Malassezia pachydermatis*, ketoconazole

## Abstract

The aim of this study was to establish a ketoconazole susceptibility test for *Malassezia pachydermatis* using modified Leeming–Notman agar (mLNA). The susceptibilities of 33 *M. pachydermatis* isolates obtained by modified CLSI M27-A3 method were compared with the results by disk diffusion method, which used different concentrations of ketoconazole on 6 mm diameter paper disks. Results showed that 93.9% (31/33) of the minimum inhibitory concentration (MIC) values obtained from both methods were similar (consistent with two methods within 2 dilutions). *M. pachydermatis* BCRC 21676 and *Candida parapsilosis* ATCC 22019 were used to verify the results obtained from the disk diffusion and modified CLSI M27-A3 tests, and they were found to be consistent. Therefore, the current study concludes that this new novel test—using different concentrations of reagents on cartridge disks to detect MIC values against ketoconazole—can be a cost-effective, time-efficient, and less technically demanding alternative to existing methods.

## 1. Introduction

*Malassezia* spp. are zoonotic pathogens that cause atopy eczema in humans and otitis externa in dogs. *Malassezia pachydermatis* is the most common strain [1]. To inhibit the infection of *Malassezia* spp., antifungal agents, such as nystatin, ketoconazole, and intraconazole, are often used. A survey conducted in a 2007 North American Veterinary Dermatology conference found that half of the attending veterinarians had encountered drug-resistant cases of yeast infection in canine otitis [2]. A 2011 report using Epsilometer test and modified M27-A2 methods mentioned that one strain of *M. pachydermatis* showed a higher resistance against ketoconazole [3]. Some later reports have also suggested that drug-resistant isolates might have emerged from *Malassezia pachydermatis* [4,5].

Currently, there is no standard method to determine the drug susceptibility of *Malassezia pachydermatis* [6]. Modified CLSI M27-A3 method has been used with various mediums, such as RPMI 1640, Leeming–Notman, high-lipid Dixon, or urea. However, these mediums are difficult to use for *Malassezia pachydermatis* due to their high lipid content [7,8,9,10,11,12,13,14,15]. M27-A3 also requires highly skilled technicians to ensure a consistent result. Epsilometer test has been used to determine the minimum inhibitory concentration (MIC) value in *Candida* spp., and the results were consistent with CLSI M27-A3 test [16]. However, the complexity and high costs of these two methods prevent them from being used extensively for clinical screening of *Malassezia* for drug resistance.

The aim of this study therefore was to develop an inexpensive but reliable alternative method that could produce a higher consistency than those methods described above, screen the drug-resistant strains by measuring the MIC value, and provide a reference for clinicians to treat canine otitis.

## 2. Materials and Methods

Thirty-three *M. pachydermatis* strains were tested. Thirty-two of them were collected from canine otitis cases in Animal Research Farm, National Chiayi University. These were identified using polymerase chain reaction to detect the presence of internal transcribed spacer 1 region in *Malassezia pachydermatis* [17]. A standard strain of *M. pachydermatis* BCRC 21676 was bought from Bioresource Collection and Research Center (Hsinchu, Taiwan) and was used in the current study as a quality control in every test to ensure the experiment results were consistent and comparable. A standard isolate of *Candida parapsilosis* ATCC 22019, which was kindly shared by Fungal Laboratory, Department of Biochemical Science and Technology, National Chiayi University, was used in this study to control the quality of the drug continuous dilution accuracy so that the skills of the technician and the quality of commercially available drugs could not affect the results of this experiment. Modified CLSI M27-A3 test and disk diffusion test using self-made continuous drug dilution disks were conducted. Results obtained from both tests were compared.

Isolates were cultured on a modified Leeming–Notman agar (mLN agar: 1% peptone, 1% glucose, 0.2% yeast extract, 0.8% ox bile, 1% glycerol, 0.05% glycerol monostearate, 0.5% Tween 60, 2% olive oil, 1.5% agar, and 0.4% chloramphenicol). Powdered ketoconazole (Union Chemical & Pharmaceutical Co., Ltd., Taiwan, 99.999% potency) was dissolved in dimethyl sulfoxide (DMSO; Sigma^®^, Darmstadt, Germany) to required concentrations. After being filtered through a membrane filter (Minisart^®^, Darmstadt, Germany), the solution was either used immediately or stored at −80 °C.

To conduct the modified CLSI M27-A3 test [9], *M. pachydermatis* was first mixed in a Leeming–Notman broth (1% peptone, 1% glucose, 0.2% yeast extract, 0.8% ox bile, 1% glycerol, 0.05% glycerol monostearate, 0.5% Tween 60, 2% olive oil and 0.4% chloramphenicol) to a 1.0 McFarland. Different concentrations of drugs were 10-fold diluted in Leeming–Notman broth to a range of 640 μg/mL to 0.3 μg/mL as a working solution. Then, 0.1 mL of each of the above concentrations of drugs were placed in separate 2 mL centrifuge tubes. In the control group, 0.1 mL of Leeming–Notman medium was added instead. In each centrifuge tube, 0.9 mL of *M. pachydermatis* (1.0 McFarland) was added, then vortexed for 10 s. The final effective concentration of the drug ranged from 64 to 0.03 μg/mL. All mixed solutions (1 mL) were then incubated for 48 to 72 h at 35 °C until colonies were observed [9,16]. Double repeating trials were performed. If an inconsistent result was observed, the test was repeated until consistent data were obtained.

**Figure 1:**
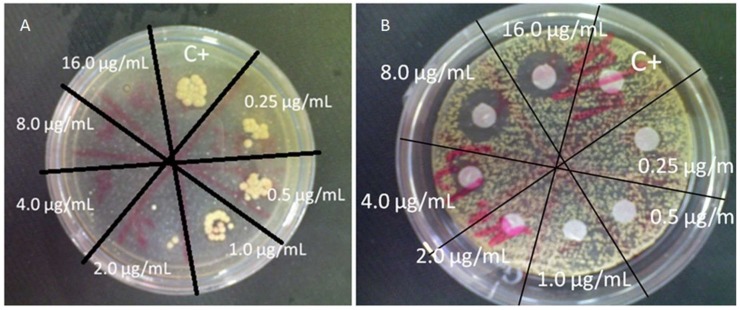
Two tests for *M. pachydermatis* drug susceptibility. (**A**) The modified M27-A3 test using Leeming–Notman agar: The minimum inhibitory concentration (MIC) values were interpreted as the minimum drug concentrations that clearly inhibits the growth of *Malassezia pachydermatis*. The number of colonies at the concentration of 2.0 μg/mL was clearly smaller than the control group. Thus, the MIC value was determined as 2.0 μg/mL. (**B**) The disk diffusion test: The MIC values were indicated when the inhibition circle began to form in the minimum drug concentrations. The inhibition circle began to form at the concentration of 4.0 μg/mL. Therefore, the MIC value was determined as 4.0 μg/mL.

In the disk diffusion test, the antifungal agent was diluted to a final working solution of 64.0 μg/mL to 0.03 μg/mL. Whatman^®^ No. 1 filter paper (0.18 mm height, Sigma^©^, Darmstadt, Germany) was used to make paper disks of 6 mm diameter. Prepared disks were pinned and sterilized in an autoclave. 1.0 McFarland Standards of *M. pachydermatis* was inoculated on the surface of mLN agar with a sterilized cotton swab and incubated at 35 °C for 30 min. 5 μL of antifungal agents with different concentrations ranging from 64.0 to 0.03 μg/mL were put on separate blank discs on the surface of a mLN agar. Five microliters of Leeming–Notman broth was added into the control group. The plates were then incubated at 35 °C for 48 consecutive hours for later interpretation. The minimum concentration that inhibits the growth of *M. pachydermatis* around each disk on the agar plate was recorded (Figure 1). Values obtained from the two tests were compared.

## 3. Results

The MIC value of *Candida parapsilosis* ATCC 22019 against ketoconazole was the same as the value suggested by CLSI 2008 using the CLSI M27-A3 technique (0.5 μg/mL) [9]. This means the ketoconazole purchased from the company, the equipment, the reagents used in this study, and the skills of the technician were functioning. Results were recorded when the MIC value of *M. pachydermatis* BCRC 21676 was 4.0 μg/mL.

Thirty-three isolates of *M. pachydermatis* (including the standard BCRC21676) were tested by the disk diffusion test and the modified M27-A3 test. The results of the isolates are listed on Table 1. In total, seven isolates had a MIC value higher than 16 μg/mL, and 16 isolates had a MIC value lower than 2 μg/mL.

Overall, 22 out of 33 isolates had the same results, and the results from the 33 isolates were very similar (Table 1). The consistency was 93.9%, and the results recorded within 2 dilutions were treated as equal. In the drug susceptibility test, in all 33 isolates using the modified CLSI M27-A3 test, the MIC values ranged from 0.5 to 16.0 μg/mL. There were two isolates with MIC values = 2.0 μg/mL; nine isolates with MIC values = 2.0 μg/mL; six isolates with MIC values = 4.0 μg/mL; two isolates with MIC values = 8.0 μg/mL; and five isolates with MIC values = 16.0 μg/mL. In the disk diffusion test, MIC values ranged from 2.0 to 16.0 μg/mL. There were 16 isolates with MIC values = 2.0 μg/mL; seven isolates with MIC values = 4.0 μg/mL; three isolates with MIC values = 8.0 μg/mL; and seven isolates with MIC values = 16.0 μg/mL.

## 4. Discussion

The main purpose of this study was to establish whether a ketoconazole susceptibility test for *Malassezia pachydermatis* using modified Leeming–Notman agar could be an alternative to the expensive and complicated M27-A3 method we currently use. Despite using commercially available drugs, the drug susceptibility test results using disk diffusion were fairly similar to those with M27-A3. Considering the MIC values were mostly within the reference range (93.3% or 31/33), this disk diffusion method using modified Leeming–Notman agar could be a cheaper, faster, and more convenient substitute for active pharmaceutical ingredient.

The mechanism of azoles is to inhibit the synthesis of 14 alpha-demethylase, which could result in no ergosterol formation [18,19]. As ergosterol is the main component of the fungal cell membrane, the malfunction of the structure of the cell membrane will result in the death of fungi [18,19]. Therefore, this study was focused on the minimum drug concentration that would cause the death of *M. pachydermatis*. Once a clear inhibition circle is formed around the disks, as long as it is clearly visible, it would be acknowledged as the concentration of drug with the effect of killing *M. pachydermatis*, regardless of the width of the inhibition circle. The drug concentration would then be recorded as the MIC value.

When the bacterial solution was inoculated onto the mLN agar with a sterile cotton swab, the number of bacteria could be controlled by the thickness of one layer of cells to avoid the difficulty of interpretation. Otherwise, inconsistent results might be observed. As colonies would form tightly around the diffusion disks, this method did not need to consider the size of the inhibition circle and only needed to visually identify the death of the strain found around the disks.

This study used two standard strains—*Candida parapsilosis* ATCC 22019 and *M. pachydermatis* BCRC 21676—for quality control. *C. parapsilosis* ATCC 22019 was used as the drug quality control, while *M. pachydermatis* BCRC 21676 was used as the sample control. The results showed that the MIC value of *C. parapsilosis* ATCC 22019 against ketoconazole measured in two different ways were within the reference range. Thus, it further confirmed the consistency of the MIC values measured by the two methods, and it had acceptable reproducibility.

While most of the MIC values were within the range, there were still inconsistencies in the MICs results of the two methods. MIC values could have been affected by solution concentration, medium variety, interacting time between drug and solution, quality of drugs, or when the reading was done. In theory, solutions with a concentration lower than MIC are unlikely to kill more bacteria. In this study, *Candida parapsilosis* ATCC 22019 was used as the drug control, and results were only recorded when they were within the reference range. Readings done at 48 and 72 h showed no difference either. It has been suggested that mediums rich in Ca^2+^ or Mg^2+^ could reduce the effectiveness of azoles drugs [15]. The mLN agar used in this study contained store-bought OMEGA rich olive oil (Uni-President, Tainan, Taiwan), which could have affected the performance of ketoconazole and caused different MICs. Future studies should try to use a medium with lower cation and higher lipid, or try to remove the 2^+^ cation by chelates, to find out whether MIC values change accordingly. It is also worth considering whether *M. pachydermatis* in Taiwan have a higher drug tolerance. Comparison with results on overseas *M. pachydermatis* isolates could offer more clear answers on this matter.

As there are still no recognized standards for MIC values in current methods, it was impossible to determine the presence or absence of drug resistance. However, if enough MIC values could be established, more could be done to compare MIC values or to consider the possibility of encountering drug-resistant *Malassezia* strains. Further research is needed on a standard procedure to test the drug susceptibility of *Malassezia* spp. and to interpret MIC values.

## Figures and Tables

**Table 1:** MIC values of microbial susceptibility test using ketoconazole disks and modified M27-A3 test.

Samples	Ketoconazole Disks (μg/mL)	Modified M27-A3 (μg/mL)
*M. pachydermatis* (BCRC 21676)	4.0	4.0
*Candida parapsilosis* (ATCC 22019)	0.5	0.5
1	2.0	2.0
2	2.0	1.0
3	2.0	0.5
4	2.0	2.0
5	4.0	2.0
6	2.0	2.0
7	2.0	2.0
8	4.0	4.0
9	2.0	4.0
10	2.0	2.0
11	4.0	4.0
12	4.0	2.0
13	16.0	16.0
14	2.0	4.0
15	2.0	2.0
16	8.0	4.0
17	8.0	8.0
18	2.0	2.0
19	2.0	4.0
20	2.0	8.0
21	16.0	4.0
22	16.0	8.0
23	8.0	4.0
24	4.0	4.0
25	4.0	4.0
26	16.0	16.0
27	16.0	16.0
28	16.0	16.0
29	16.0	16.0
30	2.0	2.0
31	2.0	2.0
32	2.0	2.0

BCRC, Bioresource Collection and Research Center; ATCC, American Type Culture Collection.
